# Emergence and spread of carbapenem-resistant *Acinetobacter baumannii* international clones II and III in Lima, Peru

**DOI:** 10.1038/s41426-018-0127-9

**Published:** 2018-07-04

**Authors:** Saúl Levy-Blitchtein, Ignasi Roca, Stefany Plasencia-Rebata, William Vicente-Taboada, Jorge Velásquez-Pomar, Laura Muñoz, Javier Moreno-Morales, Maria J. Pons, Juana del Valle-Mendoza, Jordi Vila

**Affiliations:** 1 School of Medicine, Research and Innovation Centre of the Faculty of Health Sciences, Universidad Peruana de Ciencias Aplicadas, 15067 Lima, Peru; 2 ISGlobal, Hospital Clínic – Universitat de Barcelona, 08036 Barcelona, Spain; 3 Instituto Nacional de Enfermedades Neoplásicas, 15038 Lima, Peru; 4 Hospital Nacional Arzobispo Loayza, 15082 Lima, Peru; 5 Instituto de Investigación Nutricional, 15024 Lima, Peru; 6 Present Address: Laboratorio de Microbiología Molecular y Genética Bacteriana, Universidad Científica del Sur, Lima, Peru

## Abstract

Carbapenem-resistant *Acinetobacter baumannii* is the top-ranked pathogen in the World Health Organization priority list of antibiotic-resistant bacteria. It emerged as a global pathogen due to the successful expansion of a few epidemic lineages, or international clones (ICs), producing acquired class D carbapenemases (OXA-type). During the past decade, however, reports regarding IC-I isolates in Latin America are scarce and are non-existent for IC-II and IC-III isolates. This study evaluates the molecular mechanisms of carbapenem resistance and the epidemiology of 80 non-duplicate clinical samples of *A. baumannii* collected from February 2014 through April 2016 at two tertiary care hospitals in Lima. Almost all isolates were carbapenem-resistant (97.5%), and susceptibility only remained high for colistin (95%). Pulsed-field gel electrophoresis showed two main clusters spread between both hospitals: cluster D containing 51 isolates (63.8%) associated with sequence type 2 (ST2) and carrying OXA-72, and cluster F containing 13 isolates (16.3%) associated with ST79 and also carrying OXA-72. ST2 and ST79 were endemic in at least one of the hospitals. ST1 and ST3 OXA-23-producing isolates were also identified. They accounted for sporadic hospital isolates. Interestingly, two isolates carried the novel OXA-253 variant of OXA-143 together with an upstream novel insertion sequence (IS*Aba47*). While the predominant *A. baumannii* lineages in Latin America are linked to ST79, ST25, ST15, and ST1 producing OXA-23 enzymes, we report the emergence of highly resistant ST2 (IC-II) isolates in Peru producing OXA-72 and the first identification of ST3 isolates (IC-III) in Latin America, both considered a serious threat to public health worldwide.

## Introduction

*Acinetobacter baumannii* is an opportunistic nosocomial pathogen responsible for a broad range of nosocomial infections^1^, including ventilator-associated pneumonia and bacteremia (35–52% mortality)^1,2^, as well as skin and soft tissue infections, endocarditis, urinary tract infections, and meningitis^1,3^. Nosocomial isolates of this bacterium are often resistant to most currently available antibiotics. Carbapenem-resistant *A. baumannii* has recently been considered the most critical pathogen for public health, topping the global priority list of antibiotic-resistant bacteria published by the World Health Organization^4^. *Acinetobacter baumannii* strains can develop resistance to all the antibiotics available^5^. Outbreaks caused by multidrug-resistant (MDR), extensively drug-resistant (XDR), and even pan-drug-resistant (PDR) strains having been reported worldwide^6–9^. In the past decade, resistance rates have been rising, and recent reports show a steady increase in carbapenem resistance among *A. baumannii* strains^8,10–13^.

Resistance to carbapenems in *A. baumannii* is usually mediated by the expression of carbapenem-hydrolyzing class D β-lactamases, also known as OXA-type carbapenemases, although the expression of different class B metallo-β-lactamases (MBLs), or even *Klebsiella pneumoniae* carbapenemase (KPC) enzymes, has also been reported^5^. The OXA-type enzymes described in *A. baumannii* belong to six different families, namely the intrinsic OXA-51 oxacillinase family, which is usually chromosomally encoded but has rarely been reported in plasmids^14^, and the acquired OXA-23, OXA-24, OXA-58, OXA-143, and OXA-235 families^5^. Although the population structure of *A. baumannii* strains is quite diverse, there seems to be a clonal spread of a few epidemic lineages that predominate over the rest^15^. In particular, the international clones I–III account for most *A. baumannii* infections worldwide and are usually associated with the production of OXA-23-like, OXA-24-like, or OXA-58-like enzymes^15^.

Our knowledge of the epidemiology and antibacterial susceptibility profiles of *A. baumannii*, however, is still incomplete in many parts of the world, including many countries in Latin America. The present study was designed to evaluate the phenotypic resistance patterns, the presence of carbapenem resistance mechanisms, and the clonal relatedness of *A. baumannii* isolates circulating in Lima, Peru.

## Results

### Bacterial isolates

A total of 80 non-redundant *A. baumannii* isolates were recovered from blood (*n* = 59, 73.8%), bronchial aspirate (*n* = 15, 18.8%), soft tissue (*n* = 2, 2.5%), cerebrospinal fluid (*n* = 2, 2.5%), and urine samples (*n* = 2, 2.5%) of different patients admitted to two tertiary care hospitals in Lima. Fifty-three isolates were recovered at the Instituto Nacional de Enfermedades Neoplásicas (INEN) from February 2014 through April 2016, and the remaining 27 isolates originated from inpatients at the Hospital Nacional Arzobispo Loayza (HNAL) from July through October 2015.

### Antibiotic susceptibility

Overall, antimicrobial susceptibility testing by disc diffusion reported high resistance rates to most of the antibiotics tested. More specifically, resistance to both imipenem and meropenem was as high as 97.5%, with only two isolates being susceptible to carbapenems, one from each hospital. The non-susceptibility rates to ceftazidime, cefotaxime, piperacillin/tazobactam, and levofloxacin were 98.8%, 97.5% to cefepime, 93.8% to trimethoprim-sulfamethoxazole, 80% to tetracycline, 76.3% to gentamicin, 63.8% to ampicillin/sulbactam, 62.5% to doxycycline, 61.3% to amikacin, and 57.5% to tobramycin. Susceptibility only remained high for colistin (95%); the colistin minimal inhibitory concentrations (MICs) ranged from 0.25 to 16 mg/L. The colistin MIC_50_ and MIC_90_ were 1 and 2 mg/L, respectively.

All the isolates were resistant to at least one antibiotic from three different classes. One isolate (1.2%) was resistant to all the antimicrobial agents tested and was therefore classified as PDR. Thirty-seven isolates (46.3%) presented an XDR phenotype, most being susceptible to colistin alone, and the remaining 42 isolates (52.5%) were therefore considered MDR.

Antibiotic susceptibility testing by gradient diffusion of selected strains (see below) showed elevated MIC values of carbapenems (>32 mg/L) in all the strains producing acquired class D carbapenemases as well as elevated MIC values of cephalosporins, aminoglycosides, and quinolones, in good agreement with previous data obtained by disc diffusion (Table 1). Interestingly, three strains presented MIC values of tigecycline of 4 mg/L and therefore should be considered resistant according to the European Committee on Antimicrobial Susceptibility Testing (EUCAST) breakpoints for Enterobacteriaceae^16^.

**Table 1:** Antimicrobial susceptibility and molecular characterization of selected *A. baumannii* isolates from two tertiary hospitals in Lima, Peru

Strain	Source	MIC (mg/L)											PT	ST	aOXA	iOXA
		IP	MP	CAZ	FEP	CTX	AK	GM	COL^a^	TGC	CiP	LEV				
3	INEN	>32	>32	>256	192	>32	48	>256	0.5	4	>32	>32	A	1	23	69
29	INEN	>32	>32	>256	256	>32	96	>256	1	4	>32	>32	A	1	23	69
33	INEN	>32	>32	>256	48	>32	32	2	0.5	4	>32	>32	B	79	253	65
54	HNAL	0.38	0.75	4	16	>32	96	3	1	2	>32	>32	C	79	—	65
21	INEN	>32	>32	128	16	>32	>256	>256	2	2	>32	>32	D	2	72	66
34	INEN	>32	>32	>256	24	>32	3	2	0.5	3	>32	>32	D	2	72	66
47	INEN	>32	>32	192	24	>32	>256	>256	0.5	3	>32	32	D	2	72	66
55	HNAL	>32	>32	192	24	>32	>256	>256	0.5	3	>32	>32	D	2	72	66
69	HNAL	>32	>32	>256	24	>32	>256	>256	0.5	2	>32	>32	D	2	72	66
79	HNAL	>32	>32	>256	24	>32	>256	>256	0.5	2	>32	>32	D	2	72	66
42	INEN	>32	>32	>256	24	>32	>256	>256	0.5	2	>32	>32	E	2	72	66
4	INEN	>32	>32	256	24	>32	16	>256	0.5	1.5	>32	>32	F	79	72	65
32	INEN	0.5	1	>256	24	>32	64	4	0.5	2	>32	32	F	79	—	65
56	HNAL	>32	>32	96	24	>32	32	256	1	1.5	>32	32	F	79	72	65
37	INEN	>32	>32	96	32	>32	16	0.38	1	0.25	>32	32	G	3	23	71
60	HNAL	>32	>32	96	64	>32	32	0.38	1	0.5	>32	>32	G	3	23	71
61	HNAL	>32	>32	>256	16	>32	48	>256	1	1	>32	>32	H	108	72	132
53	INEN	>32	>32	>256	32	>32	24	2	0.5	2	>32	24	I	79	253	65

*MIC* minimum inhibitory concentration, *INEN* Instituto Nacional de Enfermedades Neoplásicas, *HNAL* Hospital Nacional Arzobispo Loayza, *IP* imipenem, *MP* meropenem, *CAZ* ceftazidime, *FEP* cefepime, CTX cefotaxime, *AK* amikacin, *GM* gentamicin, *COL* colistin, *TGC* tigecycline, *CiP* ciprofloxacin, *LEV* levofloxacin, *PT* pulsotype, *ST* sequence type, *aOXA* acquired OXA-type, *iOXA* intrinsic OXA-type

^a^Colistin’s MIC was determined by broth microdilution

### Detection of carbapenem resistance genes

The presence of genes encoding both intrinsic and acquired class D carbapenemases, KPC, and MBLs was investigated by polymerase chain reaction (PCR). None of the isolates were positive for the genes encoding KPC or MBLs, but the intrinsic *bla*_OXA-51_-like gene was detected in all the samples. In two isolates, this gene was the only carbapenemase detected, although it could not be associated with the presence of an upstream IS*Aba1* element, in good agreement with the carbapenem-susceptible phenotype of these two isolates.

The *bla*_OXA-24_-like gene, however, was predominant and present in 65 isolates (81.3%) collected from both hospitals, whereas 11 isolates (13.8%) carried the *bla*_OXA-23_-like gene, and 2 isolates (2.5%) proved positive in the multiplex PCR for the *bla*_OXA-143_-like gene. Overall, there were no substantial differences between the two hospitals regarding the proportion of isolates carrying different acquired class D carbapenemases (Table 2).

**Table 2:** Number and percentages of *A. baumannii* isolates from each participating center carrying different acquired *bla*_OXA_ genes

OXA-type (acquired)	INEN		HNAL		Total	
	*N*	%	*N*	%	*N*	%
*bla*_OXA-24_	43	81.1	22	81.5	65	81.3
*bla*_OXA-23_	7	13.2	4	14.8	11	13.8
*bla*_OXA-143_	2	3.8	0	0	2	2.5
None	1	1.9	1	3.7	2	2.5
Total	53		27		80	

*N* number of isolates, *%* percentage of isolates, *INEN* Instituto Nacional de Enfermedades Neoplásicas, *HNAL* Hospital Nacional Arzobispo Loayza

### Pulsed-field gel electrophoresis

**Fig. 1:**
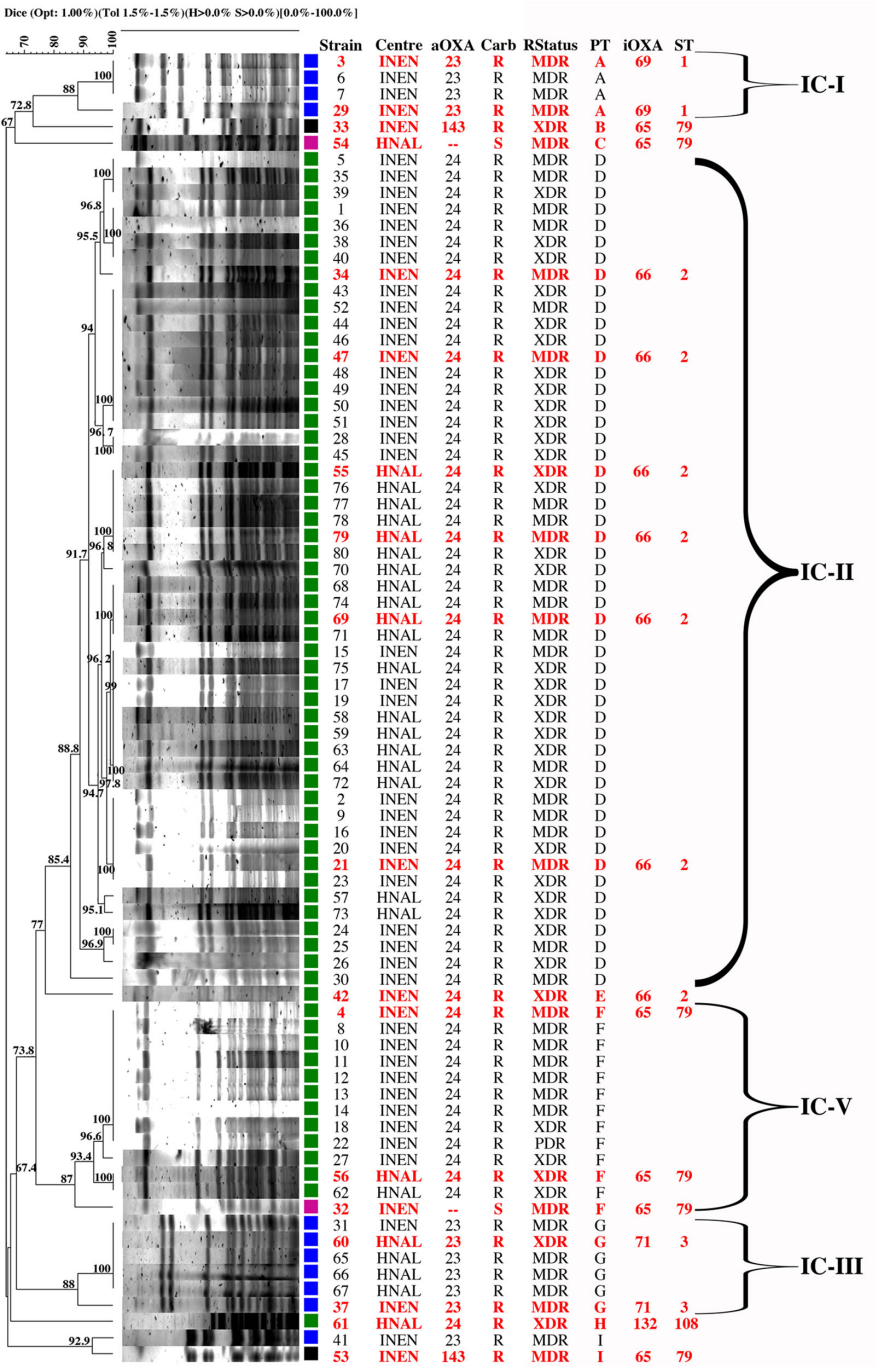
PFGE dendrogram of *A*. *baumannii* isolates from two tertiary hospitals in Lima showing carriage of intrinsic (iOXA) and acquired *bla*_OXA_ variants (aOXA); susceptibility (S) or resistance (R) to carbapenems (Carb); categorization as MDR, XDR, or PDR (RStatus); pulsotype (PT); and sequence type (ST). Isolates in red were selected as representative of each clonal group. Braces indicate classification to the corresponding international clones I–V (IC). Isolates were included in the same pulsotype if their Dice similarity index was ≥85%. Colored squares indicate production of distinct families of acquired OXA-type enzymes: blue: OXA-23; green: OXA-24; black: OXA-143; magenta: none. INEN Instituto Nacional de Enfermedades Neoplásicas, HNAL Hospital Nacional Arzobispo Loayza, MDR multidrug-resistant, XDR extensively drug-resistant, PDR; pan-drug-resistant

**Fig. 2:**
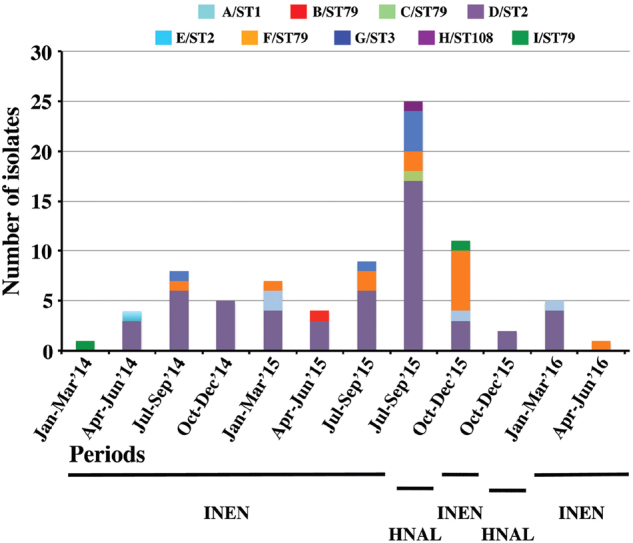
Temporal and spatial distribution of *A*. *baumannii* isolates recovered from patients at two tertiary hospitals in Lima, Peru. Stacked columns show the number of isolates from each pulsotype/sequence type (PT/ST) recovered at each institute over a 3-month period. INEN Instituto Nacional de Enfermedades Neoplásicas, HNAL Hospital Nacional Arzobispo Loayza

The clonal relatedness was initially studied by pulsed-field gel electrophoresis (PFGE), as described below, which allowed the classification of all the isolates into nine different clusters, or pulsotypes (A–I) (Fig. 1). Two major clusters disseminating between the two healthcare settings were identified: pulsotypes D and F, which contained 51 (63.8%) and 13 (16.3%) isolates, respectively. All the isolates in these two clusters were associated with the carriage of OXA-24-like carbapenemases, except for one isolate within pulsotype F with no acquired carbapenemases that was susceptible to carbapenems. OXA-23-like-producing *A. baumannii* were mostly grouped in pulsotype G (*n* = 6, 7.5%), containing isolates recovered from both hospitals, and pulsotype A, with only four isolates (5%) that were recovered from INEN. Pulsotype I contained only two isolates also recovered from INEN, but one isolate carried an OXA-23-like enzyme, while the other carried an OXA-143-like oxacillinase. Pulsotypes B, C, E, and H contained singletons (1.3%) producing OXA-24-like (pulsotypes E and H), OXA-143-like (pulsotype B), or no acquired OXA-like enzymes (pulsotype C), the latter isolate being susceptible to carbapenems. Figure 2 shows the temporal and spatial distributions of all the clones.

### Multilocus sequence typing analysis and OXA sequencing of selected strains

**Fig. 3:**
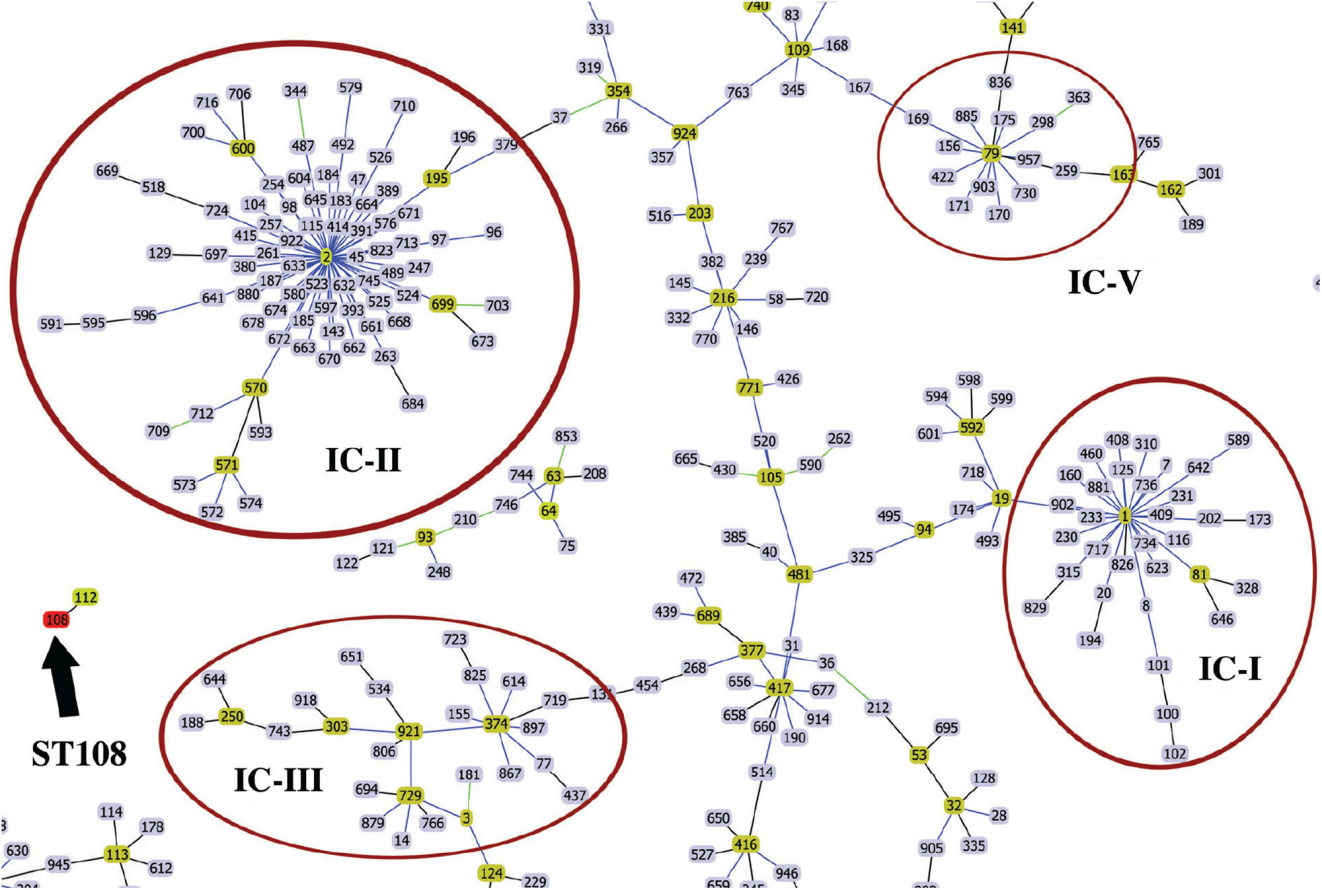
Partial snapshot of the population structure of *A*. *baumannii* according to MLST and using goeBURST (PHYLOViZ). Blue links show SLVs. Founder STs are highlighted in yellow. Reddish circles indicate isolates grouped into a particular international clone (IC). The black arrow points at ST108 and its SLV, ST112

Eighteen isolates were selected for further characterization on the grounds of their clustering in the PFGE dendrogram, susceptibility profiles, and carriage of acquired OXA-type carbapenemases (Table 1). Multilocus sequence typing (MLST) analysis using the Pasteur scheme of selected isolates showed full agreement among isolates clustered within the same pulsotype and ST designation (Table 1). Isolates from the major PFGE cluster D as well as the singleton from pulsotype E were assigned to ST2 (ST2), which belonged to clonal complex 2 (CC2) and to international clone II. Isolates from the PFGE cluster F, both isolates from pulsotype I and the singletons B and C were assigned to ST79, which was included in CC79, international clone V^17^. Isolates from pulsotype A were assigned to ST1, belonging to CC1 and international clone I, and those of pulsotype G to ST3, belonging to CC3, international clone III. The H singleton belonged to ST108, which was not assigned to any CC or international clone (Fig. 3).

Sequencing of the acquired class D oxacillinase genes in all the selected isolates revealed the presence of the OXA-72 variant in ST2, ST79, and ST108 isolates bearing a *bla*_OXA-24_-like gene as well as the presence of the novel OXA-253 variant in the two isolates with a *bla*_OXA-143_-like allele. Likewise, sequencing of the intrinsic class D oxacillinase gene identified specific OXA-51-like variants associated with each ST: ST1 isolates presented the *bla*_OXA-69_ gene, ST2 was associated with *bla*_OXA-66,_ ST3 isolates carried the *bla*_OXA-71_ gene, ST79 was linked with *bla*_OXA-65_, and ST108 had the *bla*_OXA-132_ variant (Table 1).

Of note, sequence analysis of the genetic structures surrounding the *bla*_OXA_ alleles identified the presence of the insertion sequence IS*Aba1* in reverse orientation upstream from the *bla*_OXA-23_ gene in all ST1 and ST3 isolates, although we were not able to detect the presence of a downstream IS*Aba1* copy, suggesting a Tn*2008*-like structure in these isolates^18^. Additionally, a novel insertion sequence was identified in forward orientation upstream of *bla*_OXA-253_ in the two strains carrying this OXA enzyme. To provide an attribution number to the new IS, its nucleotide sequence was submitted to ISFinder, the reference center for bacterial insertion sequences (http://www-is.biotoul.fr)^19^, and it was designated as IS*Aba47*. This novel mobile element showed 82.5% identity at the nucleotide level with IS*Aba9* and was related to the IS*982* family. The downstream region flanking *bla*_OXA-253_, however, resembled that previously described by Girlich et al^20^. A 2334 bp sequence containing the *bla*_OXA-253_ gene together with upstream and downstream flanking regions (which included IS*Aba47*) from strain 33 was also submitted to GenBank and can be retrieved under accession number MH347317.

## Discussion

In recent decades, we have witnessed the emergence of MDR *A. baumannii* isolates worldwide, which has been associated with the rapid spread of a few carbapenem-resistant epidemic lineages producing acquired OXA-type carbapenemases^1,15^. To date, however, there are only a few studies regarding the epidemiology and carbapenem susceptibility of *A. baumannii* in Latin America, and this is the first study providing such data from Peru. Interestingly, the predominant *A. baumannii* lineages reported in Latin America so far are different from those reported in other parts of the world, as international clones II and III are currently absent in this region. International clone II was reported in Brazil in the past, when ST2 isolates producing OXA-23 were described from 1999 through 2003, but it disappeared in 2004^21,22^. Instead, predominant clones are linked to ST79 (international clone V), ST25 (international clone VII), ST15, and, to a lesser extent, ST1 (international clone I), all of which are mainly associated with the production of OXA-23 enzymes^17,23–30^.

Nevertheless, there are a few reports describing OXA-72-producing *A. baumannii* isolates in Latin America, and overall, these isolates are considered less prevalent^30–33^, or at least they were until recently. In 2017, Pagano et al.^34^ described the emergence of CC79 and CC15 *A. baumannii* isolates carrying *bla*_OXA-72_ in Brazil and warned of the potential dissemination of these epidemic lineages. Likewise, Nunez Quezada et al.^35^ reported an outbreak in Ecuador in 2017 caused by OXA-72-producing *A. baumannii*^35^, although the ST of these isolates was not investigated.

The data presented in this study show extremely high resistance rates (>97%) and MIC levels (>32 mg/L) of imipenem and meropenem as well as the predominance of the MDR phenotype among *A. baumannii* isolates recovered from two tertiary hospitals in the capital city of Peru. Fortunately, the majority (95%) of isolates remain susceptible to colistin, as opposed to several studies reporting increasing rates of colistin-resistant *A. baumannii* isolates in different countries^8^. During the study period, carbapenem resistance in both settings was linked to the widespread dissemination of a major clone producing OXA-72 and belonging to ST2, the founder ST of the epidemic international clone II that has spread globally (Fig. 3)^15^. A second group of highly clonal strains producing OXA-72 and belonging to ST79, international clone V, was also identified in both hospitals. In addition, we report the presence of a few sporadic clones producing OXA-23 and linked to international clones I and III (ST1 and ST3, respectively).

International clonal lineages have traditionally been associated with the carriage of specific genetic variants of the intrinsic *bla*_OXA-51_ gene^24,28,29^. This correlation is also shown in our study and supports the genetic relatedness of these isolates with epidemic international lineages. The *bla*_OXA-65_, *bla*_OXA-66_, *bla*_OXA-69_, and *bla*_OXA-71_ genes were detected in isolates belonging to ST79, ST2, ST1, and ST3, respectively. We also identified the *bla*_OXA-132_ variant in an ST108 isolate that did not belong to any known CC. Interestingly, a single ST108 isolate carrying *bla*_OXA-132_ was reported in Lebanon in 2014, and *bla*_OXA-132_ was also present in ST15 isolates in Portugal as well as in an ST197 isolate from Saudi Arabia^27,36,37^.

The temporal and spatial distributions of the isolates (Fig. 2) show that the OXA-72-producing ST2 clone was present in both centers throughout the study period and was likely endemic in INEN, while OXA-72-producing ST79 isolates were sporadic during 2014 and also became endemic at INEN during the second half of 2015. Unfortunately, the collection period of *A. baumannii* isolates from HNAL was too short to extrapolate endemicity, but as many as 17 ST2 isolates were recovered from July through September 2015, suggesting at least an outbreak situation (Fig. 2). Patients are frequently referred between the two hospitals because INEN gathers most patients with neoplastic diseases. That might explain the presence of shared high-risk clones. Of note, 29 out of 37 XDR isolates were OXA-72-producing ST2 isolates. The single PDR isolate, however, was an OXA-72-producing ST79 isolate (Fig. 1).

We would also like to highlight the identification of two different ST79 isolates carrying an OXA-253 variant from the OXA-143 family. OXA-253 was first described from an ST79 *A. baumannii* isolate in Honduras, and to date, only a few additional studies have reported this enzyme in *A. baumannii* isolates from different STs in Brazil^20,38,39^. In addition, the genetic environment of *bla*_OXA-253_ in this study resembled that described previously by Girlich et al.^20^, except for the presence of a novel insertion sequence (IS*Aba47*) in the upstream region. IS*Aba47* might have a role in the mobilization and expression of this gene, as is already the case for the *bla*_OXA-23_, *bla*_OXA-58_, and *bla*_OXA-235_ genes encoding likewise acquired OXA-type carbapenemases that typically present different flanking IS*Aba* sequences^5^. To our knowledge, this is the first time that the presence of an IS has been reported to flank a gene belonging to the *bla*_OXA-143_ family.

In summary, while the emergence of carbapenem-resistant *A. baumannii* in Latin America has been associated with the spread of OXA-23-producing ST1, ST15, ST25, and ST79 clonal lineages^28^, the results of our study reveal the dissemination of OXA-72-producing XDR clones of *A. baumannii* in Peru as well as the identification of the epidemic international clones II and III, which were previously absent in this region. The findings presented here are extremely worrisome and should warrant the implementation of infection control measures as well as national and international surveillance measures in the region to contain the further spread of what could be considered nosocomial pandemic lineages of XDR *A. baumannii*.

## Materials and methods

### Samples

This study included 80 consecutive clinical isolates of *A. baumannii* collected from different inpatients at two tertiary care hospitals (HNAL and INEN) from February 2014 through April 2016 in Lima, Peru. Only the first isolate from each patient was included in the study. The samples were obtained from blood, bronchial aspirate, soft tissues, cerebrospinal fluid, and urine, all of which were initially identified as *Acinetobacter* spp. by the BD Phoenix™ Automated Microbiology System (BD Biosciences, USA) and later identified to the species level by matrix-assisted laser desorption ionization–time of flight mass spectrometry (Bruker, Germany) as described previously^40^.

### Antimicrobial susceptibility testing

Antimicrobial susceptibility was assessed by disc diffusion on Mueller–Hinton agar plates in accordance with the Clinical and Laboratory Standards Institute (CLSI) guidelines for the following antimicrobials: ampicillin-sulbactam, piperacillin-tazobactam, cefotaxime, ceftazidime, cefepime, gentamicin, amikacin, levofloxacin, doxycycline, tetracycline, meropenem, imipenem, and trimethoprim-sulfamethoxazole. Susceptibility to colistin was assessed by broth microdilution as recommended by the joint CLSI-EUCAST Polymyxin Breakpoints Working Group^41^. The MIC values of selected strains were also determined by gradient diffusion (*E* test, bioMérieux, Sweden) for the following antimicrobials: imipenem, meropenem, cefotaxime, ceftazidime, cefepime, gentamicin, amikacin, tigecycline, ciprofloxacin, and levofloxacin. The MICs were interpreted according to CLSI clinical breakpoints and expert rules for *Acinetobacter*^41^, except for tigecycline, which was interpreted using the EUCAST breakpoints and rules for Enterobacteriaceae (Version 8.0, January 2018)^42^. *Escherichia coli* ATCC 25922 and *A. baumannii* ATCC 19606 were used as quality control strains.

All the isolates were categorized as MDR, XDR, or PDR according to the following ad hoc definitions; MDR, non-susceptible to at least one antimicrobial agent from three classes tested; XDR, non-susceptible to all antimicrobial agents tested but two or fewer; PDR, resistant to all the antimicrobial agents tested^7^.

### Detection of carbapenem resistance genes

The presence of the following carbapenemase-encoding genes was screened by PCR: *bla*_KPC_, for serine class A carbapenemases;^43^ *bla*_NDM_, *bla*_IMP_, *bla*_VIM_, *bla*_SPM_, and *bla*_SIM_ for class B MBLs;^44^ and *bla*_OXA-51_-like, *bla*_OXA-23_-like, *bla*_OXA-24_-like, *bla*_OXA-58_-like, *bla*_OXA-143_-like, and *bla*_OXA-235_-like, for class D oxacillinases^45–47^. Amplification products were purified from agarose gels (SpinPrep^TM^ Gel DNA Kit, San Diego, CA, USA) and sent for Sanger sequencing (Macrogen, Korea) whenever necessary. The genetic identity of *bla*_OXA_ genes was determined upon pairwise sequence alignment with reference sequences retrieved from http://www.lahey.org/Studies/.

The presence of IS sequences flanking the *bla*_OXA_ genes was studied by PCR using specific primers as well as by inverse PCR and primer walking whenever needed. IS structures were annotated manually using BLASTn and the NCBI bacterial and ISFinder databases^19,48^.

### Molecular typing

PFGE was performed as described previously^11^, using genomic digestions with the *Apa*I restriction enzyme and a CHEF-DRIII system (Bio-Rad Laboratories). Molecular patterns were analyzed with InfoQuest^TM^ FP v.5.4 software (Bio-Rad Laboratories) and the unweighted pair group method with arithmetic mean to create dendrograms based on Dice’s similarity coefficient. Using bandwidth tolerance and optimization values set at 1.5 and 1%, respectively, isolates were considered to belong to the same PFGE cluster (pulsotype) if their Dice similarity index was ≥85%^49^.

MLST was performed using the Pasteur scheme for *A. baumannii*^50^. The allele sequences and STs of selected strains were identified and retrieved from the PubMLST *A. baumannii* MLST database (http://pubmlst.org/abaumannii/). The population structure of STs was evaluated using the goeBURST software (http://www.phyloviz.net/goeburst/).
